# Integrated proteomic and metabolomic modules identified as biomarkers of mortality in the Atherosclerosis Risk in Communities study and the African American Study of Kidney Disease and Hypertension

**DOI:** 10.1186/s40246-022-00425-9

**Published:** 2022-11-03

**Authors:** Linda Zhou, Aditya Surapaneni, Eugene P. Rhee, Bing Yu, Eric Boerwinkle, Josef Coresh, Morgan E. Grams, Pascal Schlosser

**Affiliations:** 1 Department of Epidemiology, Johns Hopkins Bloomberg School of Public Health, 2024 E. Monument St., Baltimore, MD 21287 USA; 2 Nephrology Division and Endocrine Unit, Massachusetts General Hospital, Boston, MA USA; 3 Department of Epidemiology, Human Genetics, and Environmental Sciences, School of Public Health, University of Texas Health Science Center at Houston, Houston, TX USA; 4 Division of Precision Medicine, Department of Medicine, New York University, New York, NY USA

**Keywords:** Chronic kidney disease, Cluster analysis, Dimensionality reduction, Metabolomics, Mortality, Proteomics

## Abstract

**Background:**

Proteins and metabolites are essential for many biological functions and often linked through enzymatic or transport reactions. Individual molecules have been associated with all-cause mortality. Many of these are correlated and might jointly represent pathways or endophenotypes involved in diseases.

**Results:**

We present an integrated analysis of proteomics and metabolomics via a local dimensionality reduction clustering method. We identified 224 modules of correlated proteins and metabolites in the Atherosclerosis Risk in Communities (ARIC) study, a general population cohort of older adults (*N* = 4046, mean age 75.7, mean eGFR 65). Many of the modules displayed strong cross-sectional associations with demographic and clinical characteristics. In comprehensively adjusted analyses, including fasting plasma glucose, history of cardiovascular disease, systolic blood pressure and kidney function among others, 60 modules were associated with mortality. We transferred the network structure to the African American Study of Kidney Disease and Hypertension (AASK) (*N* = 694, mean age 54.5, mean mGFR 46) and identified mortality associated modules relevant in this disease specific cohort. The four mortality modules relevant in both the general population and CKD were all a combination of proteins and metabolites and were related to diabetes / insulin secretion, cardiovascular disease and kidney function. Key components of these modules included N-terminal (NT)-pro hormone BNP (NT-proBNP), Sushi, Von Willebrand Factor Type A, EGF And Pentraxin (SVEP1), and several kallikrein proteases.

**Conclusion:**

Through integrated biomarkers of the proteome and metabolome we identified functions of (patho-) physiologic importance related to diabetes, cardiovascular disease and kidney function.

**Supplementary Information:**

The online version contains supplementary material available at 10.1186/s40246-022-00425-9.

## Background

The metabolome and the proteome are inextricably linked and essential to human physiology. Proteins perform many different biological functions, from enzymatic activity to molecular transport, and metabolites are often intermediates or end-products of these reactions. Metabolites are central to energy generation and homeostasis and their concentrations are often tightly regulated through generation, transport across compartments, as well as breakdown and excretion [1–3].

Over the past decade, many publications have identified single metabolites or proteins that are associated with all-cause mortality [4–10]. For example, Hu et al. [10] identified six serum metabolites associated with all-cause mortality in chronic kidney disease. In a study of 3523 participants from the Framingham Heart Study, 38 of 85 preselected circulating protein biomarkers were associated with all-cause mortality, and the addition of proteins to a model with traditional clinical variables improved all-cause mortality prediction [4]. When evaluating larger read-outs of the metabolome and proteome; however, many biomarkers are correlated, and an integrated analysis of both metabolomic and proteomic platforms may better elucidate pathways altered early in the disease process. Together, proteins and metabolites influence and are influenced by many externally observed phenotypes, representing endophenotypes that simultaneously highlight disease relevant physiology [11]. Similarly, many diseases are characterized by de-regulated pathways rather than single metabolic reactions [12].

In this manuscript, we performed data-driven identification of pathways (modules) based on circulating proteins and metabolites in the Atherosclerosis Risk in Communities (ARIC) study and constructed aggregate measures of these modules. For this, we used Netboost, a network-analysis-based dimension reduction technique [13, 14]. In this approach, proteins and metabolites are clustered into modules based on Spearman correlation, and then module information is aggregated by a principal component analysis. We then characterized associated modules with respect to human physiology and related them to mortality. To study their relevance for CKD, we transferred the mortality-associated modules to the African American Study of Kidney Disease and Hypertension (AASK) and tested the association with mortality within this cohort of CKD patients and found in particular insulin, cardiovascular and kidney function-related modules.

## Results

### ARIC study population characteristics

The 4027 participants in the ARIC study population were an average of 76.6 years old, with 53.9% women and 17.1% African American (Table 1). In the AASK CKD cohort, there were 694 participants who were an average of 54.5 years old, with 38.5% women and 100% African American.

**Table 1:** Baseline characteristics of participants in ARIC and AASK

	ARIC^*^(N = 4046)	AASK (N = 694)
	Mean (SD)	Mean (SD)
Age, years	75.7 (5.2)	54.5 (10.7)
Woman, %	2177(53.8%)	267 (38.5%)
Black, %	695 (17.2%)	694(100%)
Heart disease, %	753 (18.6%)	352 (50.7%)
Diabetes, %	1201 (29.7%)	0 (0%)
Total Cholesterol, mg/dL	179.0 (41.7)	212.0 (45.9)
High-density lipoprotein, mg/dL	51.2 (13.8)	48.2 (16.0)
Systolic blood pressure, mmHg	130.2 (18.0)	150.9 (23.8)
Body mass index, kg/m2	28.7 (5.5)	30.5 (6.4)
Glucose, mg/dL	114.0 (28.7)	94.2(15.4)
Antihypertensive medication, %	2719 (67.2%)	694 (100%)
Glomerular filtration rate^**^, mL/min/1.73m^2^	65 (18)	46 (13)
24 h urine protein levels (mg/day)^***^	n.a	109 (41; 561)
Albumin-to-creatinine ratio (mg/g)^***^	11 (6; 23)	n.a

^*^ visit 5; ^**^ estimated GFR for ARIC and measured GFR for AASK; ^***^ Median (1st; 3rd quartile) instead of mean (SD)

### Integrated omics module formation and characterization in ARIC

**Fig. 1:**
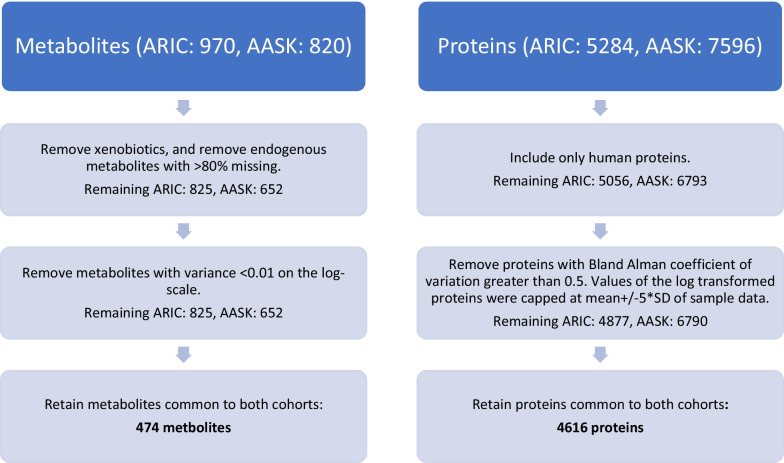
Flowchart of metabolite and protein preprocessing

**Fig. 2:**
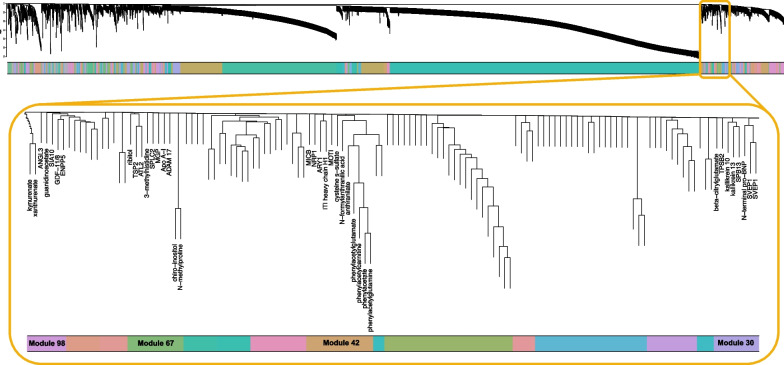
Module formation using metabolomic and proteomic data. Dendrogram of the 4616 proteins and 474 metabolites in the Atherosclerosis Risk in Communities study (ARIC). In total, 224 modules were detected. The four modules significantly associated with mortality in ARIC and AASK were zoomed in in the orange box

The 4616 proteins and 474 metabolites (Fig. 1) were clustered into 224 modules in the ARIC data (Fig. 2, Additional file 1: Table S1). There were 81 proteins and 12 metabolites that remained unassigned. The mean module size was 22.3 proteins and / or metabolites; 119 modules consisted exclusively of proteins, 61 modules consisted exclusively of metabolites, and 44 modules were a combination of proteins and metabolites. There were 371 principal components (PCs) used to represent the 224 modules (Methods).

Module PCs were related to clinical variables, demonstrating that many modules reflect a specific phenotype (Additional file 1: Table S2). For example, > 50% of the variance in the first PCs of module 25, and module 211 were explained by sex, which is consistent with many of the protein / metabolite components being hormonal regulation proteins / metabolites. The estimated glomerular filtration rate (eGFR) explained 80.5% of the variance of PC1 of module 15 and included creatinine and cystatin C among other proteins/metabolites that are known biomarkers of kidney filtration (Additional file 1: Table S2). Similarly, many of the other variables were strongly related to modules (Module 92—glucose 41.1%; module 116—high-density lipoprotein (HDL) 39.6%; module 9—total cholesterol 42.5%).

### Associations of modules with mortality

**Fig. 3:**
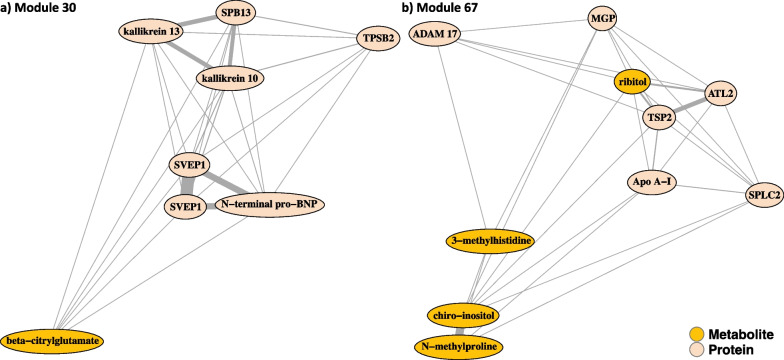
Local network structure of modules integrating metabolomic and proteomic components of prognostic significance. Edge thickness and relative distance reflect the similarity of individual components of modules

Over an average follow-up period of 6.6 years, there were 924 deaths. There were 64 module PCs that were significantly associated with mortality in ARIC in a comprehensively adjusted model (*P* < 0.05/371; Methods) representing 60 different modules (Additional file 1: Table S3). The most significant associations were module 67 PC1 (HR per SD: 1.39, *p*-value = 1.0e−16) and module 30 PC1 (HR per SD:0.74, *p*-value = 9.9e−15). The local network structures as two dimensional projections of their pairwise dissimilarities display the varying degree of linkage between the proteomic and metabolomics layers of these modules (Fig. 3). Of note, module 30 included the two aptamers of SVEP1 which are consistently highly linked. Module 67 showed that the metabolite ribitol was close to the six proteins in that module, whereas beta-citrylglutamate in module 30 was more loosely linked to a central cluster of proteins including the two SVEP1 aptamers and N-terminal pro BNP.

### Transferability of modules to AASK

**Fig. 4:**
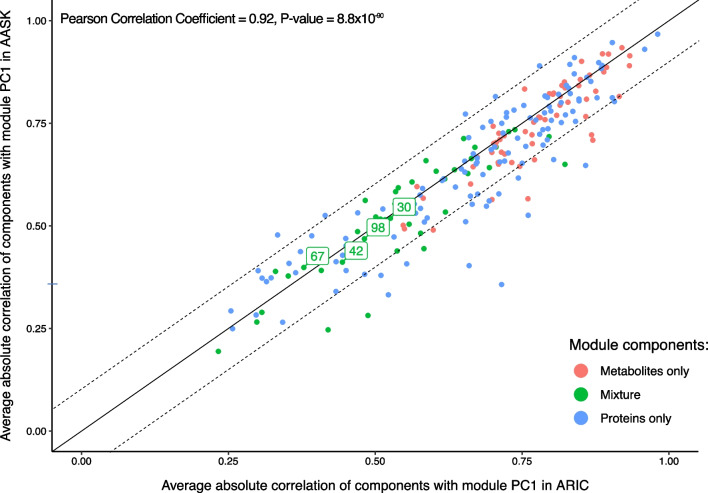
Modules from a general population cohort (ARIC) display consistent within-module correlations when transferred to a cohort of patients with CKD (AASK). Scatterplot showing transferability of the module correlation, as expressed by the correlation of components with the first module principal component, from ARIC to AASK. The overall Spearman correlation coefficient is 0.91. The four modules significantly associated with mortality in both cohorts are labeled. The diagonal and ± 0.1 offset lines are shown

**Fig. 5:**
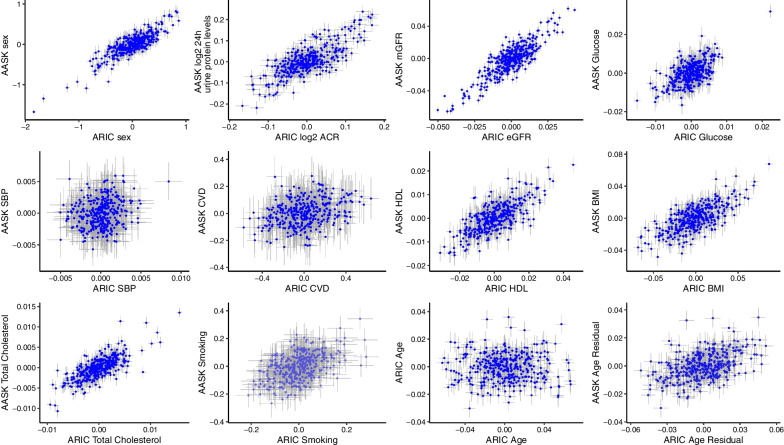
Cross-sectional associations of modules in a general population cohort (ARIC) and a cohort of patients with CKD (AASK) show many stable and strong molecular characteristics of clinical phenotypes. Graphs display the effect estimates (95% confidence interval whiskers) of linear regressions of module PCs on sex, ACR/ 24 h urine protein levels, eGFR, glucose, systolic blood pressure, history of cardiovascular disease, HDL, BMI, total cholesterol, smoking, age, and age residualized by eGFR

After transferring module membership and the PC loadings to AASK, the average Spearman correlation of module components (i.e., proteins and metabolites) to the first PC were consistent with that observed in ARIC (correlation of the average correlation coefficients, 0.91, Fig. 4). More than a third (36.2%) of the modules even had higher average Spearman correlation coefficients of proteins / metabolites with the first PC in AASK compared to ARIC, despite the PC directions being fitted on the ARIC data. Relatively few modules displayed a noticeable drop in correlation (Δcorrelation < − 0.1, 12.5%). Similarly, the regressions of module PCs on clinical traits were comparable between AASK and ARIC, particularly sex, eGFR / measured GFR (mGFR) and urinary albumin-to-creatinine ratio (ACR) / 24 h urine protein levels displayed high agreement. Notably, age displayed low transferability between the general population cohort (ARIC) and the CKD cohort (AASK) (Fig. 5). However, this appeared related to the positive association of GFR and age in AASK, an artifact of the CKD study design. Once age was adjusted for eGFR, we observed consistent correlations of age-module PCs between ARIC and AASK (Fig. 5).

In AASK, there were 148 deaths over 8.75 follow-up years. Of the 64 associations significant in ARIC 60 were direction consistent and four were also significant in AASK (*P* < 0.05/64; Table 2, Additional file 1: Table S3). All of these were mixed modules with both proteins and metabolites (Modules 30, 42, 67 and 98). The hazard ratios in AASK were consistently more pronounced than the ones in ARIC and explained a considerably proportion of risk with hazard ratios ranging from 0.61 to 1.49 per standard deviation unit.

**Table 2:** Mortality associations of the four modules associated in both the Atherosclerosis Risk in Communities study (ARIC) general population cohort and the African American Study of Kidney Disease and Hypertension (AASK) cohort of patients with chronic kidney disease

Biomarker	ARIC		AASK		Module components
Module (PC)	HR (95% CI)	*P*-value	HR (95% CI)	*P*-value	Proteins | Metabolites bold: cor_ARIC_(module PC) > = 0 non-bold: cor_ARIC_(module PC) < 0
67 (pc1)	1.39 [1.28,1.50]	1.92E−16	1.49 [1.23,1.82]	6.60E−05	ADAM 17, Apo A-I, **ATL2, MGP**, SPLC2, **TSP2 | chiro-inositol, N-methylproline, ribitol, 3-methylhistidine**
30 (pc1)	0.74 [0.68,0.80]	7.93E−15	0.61 [0.50,0.76]	4.25E−06	kallikrein 10, kallikrein 13, N-terminal pro-BNP, SPB13, SVEP1, TPSB2 | Beta-citrylglutamate
98 (pc2)	1.26 [1.17,1.36]	3.05E−9	1.49 [1.22,1.82]	1.14E−-4	**ANGL3**, ENPP5, GDF-11/8, **SIA10** | guanidinoacetate, kynurete, xanthurenate
42 (pc2)	1.2 [1.11,1.29]	1.59E−06	1.46 [1.20,1.78]	1.40E−04	ARY1, **MICB**, ITI heavy chain H1, **NRP1, MOTI, NRP1**| **anthranilate, cysteine s-sulfate**, N-formylanthranilic acid, phenylacetate, phenylacetylcarnitine, phenylacetylglutamate

Hazard ratios are in ARIC SD units

## Discussion

Metabolites and proteins are intricately linked: as substrates and enzymes, in allosteric interactions, and the assembly of protein complexes. However, few studies simultaneously evaluate the proteome and metabolome. In the present study, we integrate proteomic and metabolomic data into correlation-driven modules, demonstrate face validity through cross-sectional associations with baseline phenotypes, clinical relevance via linkage to mortality, and generalizability through transferal to a CKD cohort. We identified 60 modules of proteins and metabolites significantly associated with mortality in the general population and four of them additionally associated in the CKD cohort. As testament to the utility of combining multiple sources of omics data, all four of the modules were mixed, containing both proteins and metabolites.

We can discern specific pathological patterns associated with the four modules. For example, module 67 can be placed in the context of insulin secretion and diabetes, with many of its components associated with diabetes risk. Chiro-inositol is a secondary messenger in the insulin signaling pathway. It modulates insulin secretion, the mitochondrial respiratory chain, and glycogen storage [15]. Ribitol has been associated with diabetic retinopathy stage and was closely correlated to the module proteins in our study (Fig. 3) [16]. The protein TSP2 has been associated with levels of plasma glucose (*P* < 0.001), insulin (*P* < 0.01) and homeostasis model assessment of insulin resistance (HOMA-IR) (*P* < 0.001) by Morikawa et al. [17]. ApoA1, ApoB, and the ApoB/A1 ratio have been suggested as early indicators for predicting type II diabetes [18]. In fact, each of the module components has been implicated with insulin, risk of diabetes or both in some manner (ADAM17 [19], ATL2 [20], MGP [21], SPLC2 [22], N-methylproline [23], 3-methylhistidine [24]). Taken together, this nominates new connections between the module components and proposes module 67 as a biomarker of diabetes.

Module 30 relates to cardiovascular disease, with several of the individual components associated with hypertension and heart disease. A missense variant of the sushi, von Willebrand factor type A, EGF and pentraxin domain containing SVEP1 has been associated with coronary artery disease [25] . N-terminal pro BNP and galectin-3 are prognostic biomarkers of acute heart failure [26]. Kallikrein is active in multiple proteolytic reactions, including that of the kallikrein-kinin system and the renin-angiotensin system, and thus helps regulate blood pressure. It has been suggested that kallikrein inhibitors may have utility in the treatment of cardiovascular disease [27]. Interestingly, reduced urinary kallikrein levels have been associated with the development of high blood pressure, which is one of the major risk factors in the development of cardiac hypertrophy, ischemic heart disease, and cardiac failure [28]. Finally, the sole metabolite in module 30, beta-citrylglutamate has been associated with the single nucleotide polymorphism (SNP) rs10911021 on chromosome 1q25 and this SNP is associated with coronary heart disease in patients with type 2 diabetes [29]. Interestingly, in a recent review while some other serpins have been associated with cardiovascular pathologies SPB13 had no known pathophysiological links [30].

Module 98 and its components are related to kidney function. PC1 of module 98 showed a high correlation with GFR (cor_ARIC_ = 0.52, cor_AASK_ = 0.44; Additional file 1: Table S2). The mortality-associated PC2 of module 98 showed correlations with both sex (cor_ARIC_ = 0.4, cor_AASK_ = 0.38) and GFR (cor_ARIC_ = 0.25, cor_AASK_ = 0.26). Of its components high plasma guanidinoacetate-to-homoarginine ratio is associated with high all-cause mortality rate in adult renal transplant recipients with a hazard ratio of 1.35 [95% CI 1.19–1.53]) [31] Moreover, guanidinoacetate is very closely correlated to the proteins in the module (Additional file 1: Fig. S1). Lower kidney clearances of kynurete, a highly protein-bound solute, were associated with significantly greater risks of CKD progression [32] and has been reported to be in close association with xanthurenate [33]. ANGL3 plays a critical role in nephrotic syndrome, among several other diseases [34]. Considering the comprehensive adjustment of our mortality analyses, including sex and GFR, the module illustrates the data-driven pathway effect that goes beyond GFR-related mortality but still might reflect some form of kidney function. Notably, to our knowledge ENPP5 and GDF-11/8, the most central components of the module (Additional file 1: Table S1 and Fig. S1) have not been well studied in relation to kidney physiology.

Lastly, for module 42 we did not observe a clear pattern across all twelve components (six proteins, six metabolites). While some of the metabolites are involved in the tryptophan pathway and/or relate to kidney function (N-formylanthranilic acid, phenylacetylglutamate, anthranilate) [35], the first PC was only moderately associated with GFR (cor_ARIC_ = 0.35, cor_AASK_ = 0.30) and other components were associated with rare disorders of sulfur amino acid metabolism (cysteine s-sulfate) [36] or immune response (NRP1) [37].

A major strength of this study is the use of network methods to integrate proteins and metabolites in well-designed cohorts with large sample sizes of population and events, long follow-up, extensive metabolomics and proteomics panels, and the demonstration of transferability to an external population very different from the initial cohort. Through the unsupervised rank-based design of the network abstraction, we were able to identify data-driven pathways across the two omics domains and simultaneously structure our data and reduce the multiple testing burden. Literature review underlined the consistency of the identified modules in the endpoint associations and provided initial hypotheses with respect to their potentially shared biological pathways.

Limitations included that Netboost, similar to other correlation-based approaches, does not infer causal relations and module membership in some instances might be confounded by external influences, i.e., module members might be downstream of a common cause. Second, biological networks as reflected in proteomics and metabolomics data are complex and different network methodologies might identify different aspects of the underlying physiology. Hence, the modules inferred in our analyses should not be viewed as absolute but rather as one representation and other approaches might highlight further aspects relevant to mortality. Third, this is the first application of Netboost to proteomics data. While the approach has not been validated for this datatype, proteomics shares many of the distributional properties of metabolomics. Finally, the two cohorts are quite distinct and thus only a subset of the ARIC mortality associations was reproducible in the younger AASK CKD cohort. Whether this relates to the underlying biology or limited sample size remains to be determined. While the small sample size did limit power for the evaluation of the associations with mortality, those that do appear were among the strongest in the ARIC general population cohort and are well supported in their generalizability.

## Conclusions

This study identifies integrated biomarkers of the proteome and metabolome that relate to physiological and pathological changes important in human health and disease. We used a novel clustering technique to begin to unravel how correlated proteins and metabolites together contribute to adverse health outcomes in addition to established risk factors. Future studies are needed to explore the co-regulation of proteins and metabolites in a functional manner and to apply the findings on mortality risk with prevention and treatment in mind.

## Methods

### Study population

The ARIC study is a prospective community-based cohort of 15,792 individuals who were recruited and enrolled between 1987 and 1989 from four US communities (Forsyth County, NC; Jackson, MS; Minneapolis suburbs, MN; Washington County, MD). Details on the ARIC study design and methods have been previously published [38]. During the fifth study visit between 2011 and 2013 blood samples were collected for quantification of plasma protein and serum metabolite levels. Institutional review boards at each field center have approved of the study and written informed consent has been obtained from participants at baseline and follow-up visits. All 4046 participants with available proteomic and metabolomic profiling at visit 5 (61.6% of study visit participants) were included. The censoring date for follow-up was December 31st, 2018.

The AASK study was a trial of 1094 adult African Americans aged 18–70 years with hypertensive chronic kidney disease (mGFR 20–65 ml/min per 1.73 m^2^) recruited from 21 clinical centers in the United States. AASK trial enrollment occurred between February 1995 and September 1998, and the trial phase ended in September 2001. All 694 participants with available proteomic and metabolomic profiling at baseline in the trial phase were included in our analysis [39].

### Proteomic and metabolomic profiling

ARIC has a uniform blood collection protocol (https://sites.cscc.unc.edu/aric/Cohort_Manuals/Blood_Collection_And_Processing_7.PDF) for serum separate tubes (SST) and EDTA tubes across all 4 sites. EDTA tubes were spun (3000 g for 10 min at 4 °C) and plasma frozen. Similarly, AASK has a routine blood collection protocol for SSTs (https://repository.niddk.nih.gov/studies/aask-trial/MOOP/). In ARIC, 5282 plasma proteins were quantified in ARIC participants using a Slow Off-rate Modified Aptamer–based capture array and plasma collected at visit 5, using the SomaScan® platform v4. Similar procedures, using the expanded SomaScan® v4.1 platform, were applied to serum samples from the baseline visit in AASK, resulting in quantification of 7596 serum proteins in the AASK study [39]. For both studies, proteins were log_2_-transformed to account for skewed raw value distributions, and values outside of 5 SDs on the log_2_-scale were winsorized. In addition, we excluded proteins if the Bland Altman coefficient of variation among blind duplicate samples was greater than 0.5 (Fig. 1). The final analysis included only human proteins that were quantified in both cohorts (*N* = 4616).

Serum metabolite profiling was performed using untargeted mass spectrometry following standard protocols at Metabolon, Inc. (Morrisville, NC) using the SST samples in both studies (HD4 Platform). There were 970 and 820 metabolites of known identity quantified in the ARIC and AASK study, respectively [40]. Xenobiotics were excluded during preprocessing. Endogenous metabolites with > 80% missing was excluded. All metabolites were scaled to a median of 1 and log_2_-transformed, and metabolites with variance < 0.01 on log_2_-scales were removed. The final analysis included only metabolites that were quantified in both cohorts (*N* = 474). Missing data were imputed with minimum values (0.71% of the combined protein and metabolite analysis dataset) and capped at 5 standard deviations above or below the mean (Fig. 1).

### Module formation

Netboost is an unsupervised three-step dimension reduction technique developed in the context of DNA methylation and gene expression data [13]. In brief, first, unrelated variable pairs are filtered such that a sparse correlation-based network can be constructed on the strongest network edges. Second, variables are hierarchically clustered into modules based on the sparse network. Modules form a data-driven partition of all metabolites and proteins included in the analysis. The background module consists of 81 proteins and 12 metabolites that were left without closely related components. Third, module-aggregated measures are quantified using the PCs of each module except the background module. In this study, we used Netboost to characterize modules using combined proteomic and metabolomic data similar to previous applications to mass spectrometry data [41, 42]. The minimal module size was set to two, distance measures were based on Spearman coefficients, and robust PCs were used [13]. Highly correlated preface modules (i.e., modules with correlation of the first PCs greater than 0.9) were merged to further reduce the dimensionality. Three PCs of the modules were exported, or fewer if they already accounted for at least 50% of the module variance.

### Characterization of modules and association with mortality

After identifying modules of proteins and metabolites using Netboost in ARIC, to characterize modules we regressed module PCs on clinical traits. Clinical traits included age, sex, eGFR, ACR, HDL, body mass index (BMI), fasting plasma glucose, total cholesterol, systolic blood pressure, history of cardiovascular disease (CVD), and history of smoking. eGFR was defined using the CKD Epi 2009 equation using creatinine and cystatin C.

Next, we evaluated the associations between the module PCs and mortality using Cox proportional hazards models. Analyses were adjusted for age, sex, race-center, eGFR [43], CVD, history of smoking, diabetes, fasting plasma glucose, log 2 transformed ACR, systolic blood pressure, antihypertensive medications, HDL, total cholesterol, and BMI. Adjustment for total cholesterol and BMI used linear splines with knots at 200 mg/dL and 25 kg/m^2^, respectively [44, 45].

### Transferability of modules and relevance in a cohort with CKD

We next evaluated whether module membership transferred to a separate cohort with CKD patients. To do this, module memberships and PC loadings developed from the ARIC cohort were applied to the AASK cohort. Cross sectional regression models with the same clinical traits were used to characterize the modules and compared with those done in ARIC. To account for the AASK study design where participants were selected based on mGFR 20–65 ml/min per 1.73 m^2^, we additionally calculated correlations with age residuals from a regression on GFR.

As in ARIC, a Cox proportional hazards model was used to test for associations between the module PCs and mortality. Only those modules that had a statistically significant association with mortality in ARIC were tested in AASK. In AASK, model covariates included age, sex, mGFR, CVD, history of smoking, fasting plasma glucose, log 2 transformed 24 h urine protein levels, systolic blood pressure, HDL, total cholesterol, and BMI. Again, adjustment for total cholesterol and BMI used linear splines with knots at 200 mg/dL and 25 kg/m^2^, respectively [44, 45].

Both ARIC and AASK study analyses accounted for multiple testing by a Bonferroni adjustment for the number of analyses (*P*-value < 0.05/371 and *P*-value < 0.05/64, respectively).

## Supplementary Information

**Additional file 1**. Additional materials including the module memberships and annotations of proteins/metabolites; the cross-sectional associations of modules and participant characteristics; the mortality associations of modules; and network representations of module 42 and 98.

## Data Availability

Pre-existing data access policies for each of the parent cohort studies specify that research data requests can be submitted to each steering committee; these will be promptly reviewed for confidentiality or intellectual property restrictions and will not unreasonably be refused. Please refer to the data sharing policies of these studies on https://www2.cscc.unc.edu/aric/node/10303 (ARIC) and https://repository.niddk.nih.gov/studies/aask-trial/ (AASK).
